# Skin mottling score assesses peripheral tissue hypoperfusion in critically ill patients following cardiac surgery

**DOI:** 10.1186/s12871-024-02474-0

**Published:** 2024-04-05

**Authors:** Jing-chao Luo, Ming-hao Luo, Yi-jie Zhang, Wen-jun Liu, Guo-guang Ma, Jun-yi Hou, Ying Su, Guang-wei Hao, Guo-wei Tu, Zhe Luo

**Affiliations:** 1 Cardiac Intensive Care Center, Zhongshan Hospital, Fudan University, Shanghai, 200032 China; 2 Shanghai Geriatric Medical Center, Shanghai, 200032 China; 3 Shanghai Medical College, Fudan University, Shanghai, 200032 China

**Keywords:** Cardiac surgery, Shock, Skin mottling, Tissue hypoperfusion, Capillary refill time

## Abstract

**Background:**

Skin mottling is a common manifestation of peripheral tissue hypoperfusion, and its severity can be described using the skin mottling score (SMS). This study aims to evaluate the value of the SMS in detecting peripheral tissue hypoperfusion in critically ill patients following cardiac surgery.

**Methods:**

Critically ill patients following cardiac surgery with risk factors for tissue hypoperfusion were enrolled (*n* = 373). Among these overall patients, we further defined a hypotension population (*n* = 178) and a shock population (*n* = 51). Hemodynamic and perfusion parameters were recorded. The primary outcome was peripheral hypoperfusion, defined as significant prolonged capillary refill time (CRT, > 3.0 s). The characteristics and hospital mortality of patients with and without skin mottling were compared. The area under receiver operating characteristic curves (AUROC) were used to assess the accuracy of SMS in detecting peripheral hypoperfusion. Besides, the relationships between SMS and conventional hemodynamic and perfusion parameters were investigated, and the factors most associated with the presence of skin mottling were identified.

**Results:**

Of the 373-case overall population, 13 (3.5%) patients exhibited skin mottling, with SMS ranging from 1 to 5 (5, 1, 2, 2, and 3 cases, respectively). Patients with mottling had lower mean arterial pressure, higher vasopressor dose, less urine output (UO), higher CRT, lactate levels and hospital mortality (84.6% vs. 12.2%, *p* < 0.001). The occurrences of skin mottling were higher in hypotension population and shock population, reaching 5.6% and 15.7%, respectively. The AUROC for SMS to identify peripheral hypoperfusion was 0.64, 0.68, and 0.81 in the overall, hypotension, and shock populations, respectively. The optimal SMS threshold was 1, which corresponded to specificities of 98, 97 and 91 and sensitivities of 29, 38 and 67 in the three populations (overall, hypotension and shock). The correlation of UO, lactate, CRT and vasopressor dose with SMS was significant, among them, UO and CRT were identified as two major factors associated with the presence of skin mottling.

**Conclusion:**

In critically ill patients following cardiac surgery, SMS is a very specific yet less sensitive parameter for detecting peripheral tissue hypoperfusion.

## Background

Adequate tissue perfusion is an indispensable factor in maintaining normal metabolic processes and essential bodily functions [1]. However, in intensive care units (ICU), the occurrence of critical illnesses such as severe infection, heart failure, major trauma or surgery can disrupt this delicate balance, leading to insufficient blood flow and tissue hypoperfusion [2–6], which can result in tissue hypoxia, ischemia, and in severe cases, organ dysfunction and death. Patients undergoing major cardiac surgery, a special category of critically ill population, who are prone to bleeding, vascular vasoplegia, cardiac dysfunction, and hypotension, are particularly vulnerable to tissue hypoperfusion [6, 7]. Thus, frequent and thorough assessments are crucial in detecting early signs of tissue hypoperfusion in this population, enabling healthcare providers to intervene promptly and implement targeted interventions in real-time.

Skin mottling is a commonly observed visual characteristic of the body surface that is typically associated with peripheral tissue hypoperfusion [8]. In 2011, Ait-Oufella et al. proposed a semi-quantitative scoring system, known as the skin mottling score (SMS), to assess the extent of skin mottling [9]. This scoring system ranges from 0 to 5 and was designed to provide a more precise and accurate depiction of the severity of skin mottling. Notably, the SMS has been shown to possess a robust capacity for stratifying the risk of mortality in septic patients [9, 10]. However, the pathophysiological mechanisms that give rise to circulatory failure in critically ill patients following cardiac surgery differ significantly from those in septic shock [11]. Despite cardiogenic factors (cardiac dysfunction) [12] predominating in patients with hemodynamic instability after cardiac surgery, hypovolemia (blood loss or excessive volume restriction), distributive (vascular vasoplegia due to cardiopulmonary bypass) [13], and obstructive (pericardial effusion, pulmonary hypertension) [14] hemodynamic disturbances may all be present. Consequently, it is necessary to investigate whether the SMS performs similarly in these non-septic critically ill patients as it does in patients with septic shock.

The aim of this study was to evaluate the performance of SMS in detecting peripheral tissue hypoperfusion in critically ill patients following cardiac surgery.

## Methods

### Populations

This study conducted secondary analysis using dataset from the first phase (June 2020 to May 2021) of the NVIM cohort (Ethics Committee of Zhongshan Hospital, Fudan University, approval number B2020-057), which investigated the correlation between leg-area body surface thermal distribution and tissue perfusion in critically ill patients, incorporating numerous hemodynamic and tissue perfusion parameters [15]. Patients were assessed on the first postoperative day following admission to the ICU and enrolled patients who had risk factors of hemodynamic instability and tissue hypoperfusion, including reduced left ventricular ejection fraction (< 50%), oliguria (urine output < 0.5 ml/kg/h), tachycardia (heart rate[HR] > 100 bpm), hypotension, hyperlactatemia, prolonged capillary refill time (CRT) or cold and clammy skin and limbs. Because the leg area served as the region of interest when building the dataset, factors affecting leg surface observation or leg blood flow were excluded, including femoral artery or vein cannulation (e.g., for extracorporeal membrane oxygenation, intra-aortic balloon pump, or hemodialysis catheter placement), severe arterial abnormalities (such as stenosis, embolism, or aortic dissection), surgical incisions (e.g., in cases of coronary artery bypass graft surgery using the saphenous vein), or skin disease.

**Fig. 1:**
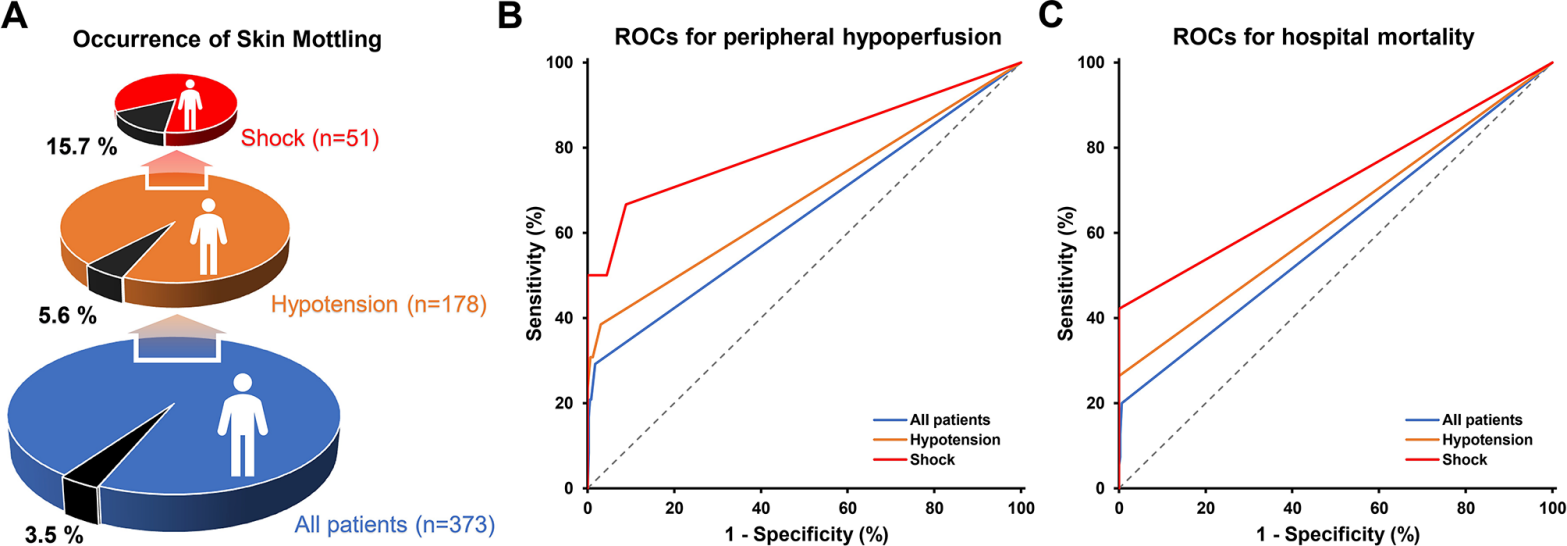
Occurrences of skin mottling in different populations (A), ROC curves for SMS to identify peripheral hypoperfusion (B) and predict hospital mortality (C) ROC: receiver operator characteristic curve

The original dataset encompassed 373 individuals, with 178 exhibiting concurrent persistent hypotension. Among this subset, an additional 51 individuals fulfilled the criteria for shock, thereby delineating three distinct populations characterized by varying degrees of severity and occurrence of skin mottling, as visually represented in Fig. 1.**A**. In this study, we sought to evaluate the utility of SMS in discerning tissue hypoperfusion across these three populations of escalating severity.

### Assessment of skin mottling

In this study, a semi-quantitative assessment of skin mottling was performed using the SMS system [9]. This system employs a scoring scheme ranging from 0 to 5, with a score of 0 indicating the absence of mottling, while scores 1 through 5 represent increasing levels of mottling severity. Specifically, a score of 1 indicated the presence of mottling only in the center of the knee, while a score of 2 indicated that it had extended to the edge of the kneecap. A score of 3 indicated mottling above the knee but not extending beyond the mid-thigh and calf, while a score of 4 indicated its spread to the ends of the thigh and calve. Finally, a score of 5 represented mottling that had spread to the groin and ankle.

### Data collection

Conventional hemodynamic and tissue perfusion parameters including SBP, diastolic blood pressure (DBP), mean arterial pressure (MAP), pulse pressure (PP), HR, UO, fingertip CRT (Syringe piston compression method [16], make three measurements and take the average), lactate, lactate change over 2 h (Δlactate), central venous oxygen saturation (ScvO_2_), venous-to-arterial carbon dioxide partial pressure difference (ΔPCO_2_) and vasoactive inotropic score (VIS) [17] were obtained at the same time as the skin mottling assessment. Follow-up was conducted until participants were either discharged or deceased.

### Definitions

The primary outcome of this study was peripheral tissue hypoperfusion, which was defined as significant prolonged CRT (> 3.0 s) [18]. The secondary outcome was hospital mortality. Skin mottling was attributed to an SMS score of 1–5, whereas no skin mottling was represented by a score of 0. Hypotension was defined as SBP < 90 mmHg or dependence on vasopressors (epinephrine, epinephrine or vasopressin) for at least one hour. Shock was defined as hypotension combined with hyperlactatemia (lactate levels ≥ 4 mmol/L [19]).

### Statistical analysis

Data were reported using the median with interquartile range (IQR) or total numbers with percentages, and statistical comparisons were made using appropriate methods, such as the Wilcoxon rank-sum test or Fisher’s exact test. The receiver operating characteristic (ROC) curves were generated to evaluate the accuracy of SMS in detecting peripheral hypoperfusion or predicting hospital mortality. The corresponding areas under the ROC curves (AUROC) were computed. Sensitivity, specificity, positive and negative predictive values (PPV and NPV) were derived at the optimal threshold established by means of the Youden’s index. Correlations between SMS and conventional hemodynamic perfusion parameters were assessed. Logistic regression (with best subset method) was employed to identify factors associated with the occurrence of skin mottling. All statistical tests were two-tailed, and statistical significance was set at a P value of < 0.05. R version 4.2.2 (R Foundation for Statistical Computing, Vienna, Austria) was utilized for all statistical analyses.

## Results

### Characteristics of patients

The study enrolled a total of 373 patients, of whom 233 (63%) were male, and the median age was 62 years (IQR: 54–69). Among the study population, 305 patients (81.8%) underwent valve surgeries, 35 patients (9.4%) underwent aortic root surgeries, 19 patients (5.1%) underwent left ventricular outflow tract surgeries, and 14 patients (3.8%) underwent other types of surgeries. Besides, 356 (95.4%) remained on mechanical ventilation support, with a median duration of 2 days (IQR: 1–6). Postoperatively, 292 patients (78.3%) received blood transfusions. Additionally, 253 patients (67.8%) had a postoperative LVEF less than 50%, and 165 patients (44.2%) received vasopressor support, with 39 patients (10.5%) specifically receiving vasopressin support.

### Patients with skin mottling

Among the total 373 patients in the dataset, 13 (3.5%) patients presented with skin mottling (Fig. 1.**A**). The severity of skin mottling varied among patients, with 5, 1, 2, 2, and 3 patients having SMS score ranging from 1 to 5, respectively. In comparison to patients without skin mottling, those with skin mottling demonstrated lower SBP (45 vs. 57 mmHg, *p* = 0.013), MAP (65 vs. 73 mmHg, *p* = 0.049), and UO (0.1 vs. 1.2 ml/kg/h, *p* < 0.001) (Table 1). They also had a higher HR (100 vs. 87, *p* = 0.042), lactate (6.0 vs. 2.2 mmol/L, *p* = 0.001), Δlactate (1.0 vs. 0 mmol/L, *p* = 0.001), VIS (32 vs. 7 µg/kg/min, *p* = 0.015), and CRT (3.1 vs. 1.2 s, *p* < 0.001) (Table 1). In the subpopulation of 178 cases of hypotension, the occurrence of skin mottling was higher, at 5.6% (Fig. 1.**A**). In addition, in the more severe population of 51 shock patients, the occurrence of skin mottling increased significantly to 15.7% in the 51 shock patients (Fig. 1.A).

**Table 1:** Comparison of clinical characteristics of patients with and without skin mottling

	All patients (*n* = 373)	No Mottling (*n* = 360)	Mottling (*n* = 13)	*p* value
Gender, n (%)	233 (62.5)	225 (62.5)	8 (61.5)	1.000
Age, year	62 (54–69)	62 (53–69)	66 (62–69)	0.141
BMI, kg/m^2^	23 (20–25)	23 (21–25)	24 (20–27)	0.570
SBP, mmHg	110 (98–124)	110 (98–124)	96 (79–125)	0.200
DBP, mmHg	57 (49–66)	57 (50–66)	45 (38–56)	0.013
MAP, mmHg	72 (63–82)	73 (64–82)	65 (55–73)	0.049
PP, mmHg	53 (43–65)	53 (43–65)	52 (39–60)	0.825
HR, bpm	87 (79–102)	87 (79–101)	100 (90–118)	0.042
CVP, mmHg	12 (10–13)	12 (10–13)	13 (11–15)	0.293
UO, ml/kg/h	1.2 (0.8–1.8)	1.2 (0.8–1.8)	0.1 (0–0.4)	< 0.001
Lactate, mmol/L	2.2 (1.5–3.8)	2.2 (1.4–3.6)	6.0 (2.9–9.4)	0.001
ΔLactate, mmol/L	0 (-0.5 to 0.2)	0 (-0.5 to 0.2)	1.0 (0.2 to 2.4)	0.001
ΔPCO_2_, mmHg	7.5 (5.2–9.7)	7.5 (5.3–9.7)	5.6 (4.2–11.1)	0.417
CRT, second	1.2 (0.9–1.8)	1.2 (0.9–1.7)	3.1 (2.4–5.6)	< 0.001
ScvO_2_, %	70 (64–75)	70 (64–75)	63 (51–75)	0.220
VIS, µg/kg/min	8 (2–20)	7 (2–19)	32 (11–53)	0.015
LOMV, day	2 (1–6)	2 (1–6)	6 (1–12)	0.176
LOICU, day	5 (2–12)	5 (2–11)	10 (2–19)	0.388
LOHS, day	16 (12–23)	17 (12–23)	15 (11–23)	0.525
Mortality, n (%)	55 (14.7)	44 (12.2)	11 (84.6)	< 0.001

BMI: body mass index; SBP: systolic blood pressure; DBP: diastolic blood pressure; MAP: mean arterial pressure; PP: pulse pressure; HR: heart rate; CVP: central venous pressure; UO: urine volume; ΔLactate: lactate change over 2 h; CRT: capillary refill time; ScvO_2_: central venous oxygen saturation; ΔPCO_2_: venous-to-arterial carbon dioxide partial pressure difference; VIS: vasoactive inotropic score; LOMV: length of mechanical ventilation; LOICU: length of intensive care unit; LOHS: length of hospital stay

Data are presented as median (interquartile range) or count (percentage)

### The performance of SMS in detecting peripheral hypoperfusion

Tables 2** and** Fig. 1.**B** demonstrated the diagnostic performance of SMS for peripheral hypoperfusion. Of the total population of 373 cases, 24 (6.4%) patients had a significant prolonged CRT. The AUROC of SMS in detecting peripheral hypoperfusion was 0.64 (95% CI: 0.59–0.69). The optimal threshold of SMS was 1, corresponding to sensitivity, specificity, PPV, and NPV of 29 (95% CI: 13–51), 98 (95% CI: 96–99), 54 (95% CI: 25–81), and 95 (95% CI: 93–97), respectively. In the hypotensive population, 13 (7.3%) patients had peripheral hypoperfusion and the AUROC was mildly elevated to 0.68 (95% CI: 0.61–0.75), corresponding to an increase in sensitivity to 38 (95% CI: 14–68). In the shock population, the proportion of patients with peripheral hypoperfusion increased to 11.8% and the corresponding AUROC and sensitivity increased substantially to 0.81 (95% CI: 0.673–0.90) and 67 (95% CI: 22–96), while the specificity remained very high at 91 (95% CI: 79–98). We also note that the NPV consistently stayed above 90, while the PPV hovered at a low level of 50, regardless of how the population was defined.

**Table 2:** The accuracy of SMS for detecting peripheral hypoperfusion or predicting hospital mortality

	For peripheral hypoperfusion			For hospital mortality		
Population	All patients	Hypotension	Shock	All patients	Hypotension	Shock
AUROC	0.64 (0.59–0.69)	0.68 (0.61–0.75)	0.81 (0.673–0.90)	0.60 (0.55–0.65)	0.63 (0.56–0.70)	0.71 (0.57–0.83)
Cutoff	1	1	1	1	1	1
Sensitivity	29 (13–51)	38 (14–68)	67 (22–96)	20 (10–33)	26 (13–43)	42 (20–67)
Specificity	98 (96–99)	97 (93–99)	91 (79–98)	99 (98–100)	100 (97–100)	100 (89–100)
PPV	54 (25–81)	50 (19–81)	50 (16–84)	85 (55–98)	100 (69–100)	100 (63–100)
NPV	95 (93–97)	95 (91–98)	95 (84–99)	88 (84–91)	83 (75–87)	74 (59–86)

SMS: skin mottling score; AUROC: area under the receiver operating characteristic curve; PPV: positive predictive value; NPV: negative predictive value

Data are presented as values and their 95% confidence intervals

### Correlation among SMS and conventional perfusion parameters

Table 3 described the correlation coefficients between conventional perfusion parameters for SMS as well as odds ratios (OR) of conventional perfusion parameters for the presence of skin mottling. For the whole population, significant correlations were found for UO, lactate, CRT and VIS and SMS with correlation coefficients of -0.19, 0.28, 0.5 and 0.39 respectively. For subpopulations with hypotension or shock, the correlation coefficients between the most of above parameters and SMS tended to increase. According to univariate logistic regression, all parameters except Δlactate were associated with the risk of developing skin mottling. The best subset method identified UO and CRT as the perfusion parameters most closely associated with the presence of skin mottling, with adjusted ORs of 0.03 (95% CI: 0.01–0.17) and 2.15 (95% CI: 0.02–0.41), respectively. The combination of these two parameters determined the appearance of skin mottling with a C-index of 0.93 (95% CI: 0.86–1.00).

**Table 3:** Correlation of conventional circulatory parameters with SMS

Population	Parameters	MAP	UO	Lactate	Δlactate	ΔPCO_2_	CRT	ScvO_2_	VIS
All patients	ρ with SMS	-0.11	-0.19	0.28	0.11	-0.03	0.50	-0.09	0.39
	p value	0.298	< 0.001	< 0.001	0.052	0.578	< 0.001	0.092	< 0.001
	OR for mottling	0.95 (0.90–0.99)	0.03 (0.01–0.17)	1.28 (1.14–1.44)	2.35 (1.50–3.67)	1.02 (0.95–1.09)	2.15 (1.54–2.99)	0.95 (0.92–0.99)	1.03 (1.01–1.05)
	p value	0.027	< 0.001	< 0.001	< 0.001	0.649	< 0.001	0.019	0.008
Hypotension	ρ with SMS	-0.21	-0.22	0.40	0.15	-0.01	0.61	-0.15	0.50
	p value	0.004	0.002	< 0.001	0.046	0.973	< 0.001	0.049	< 0.001
	OR for mottling	0.92 (0.86–0.98)	0.01 (0–0.17)	1.32 (1.14–1.53)	2.18 (1.36–3.5)	1.03 (0.96–1.11)	1.9 (1.36–2.65)	0.94 (0.90–0.98)	1.02 (1.00–1.05)
	p value	0.009	0.002	< 0.001	0.001	0.361	< 0.001	0.008	0.045
Shock	ρ with SMS	-0.32	-0.36	0.39	0.27	-0.14	0.67	-0.13	0.02
	p value	0.023	0.010	0.005	0.060	0.339	< 0.001	0.355	< 0.001
	OR for mottling	**0.90 (0.82–0.99)**	**0.01 (0–0.48)**	**1.22 (1.00–1.48)**	**1.96 (1.18–3.25)**	1.00 (0.91–1.11)	**1.73 (1.10–2.71)**	0.96 (0.91–1.01)	1.02 (0.99–1.04)
	p value	0.025	0.018	0.046	0.009	0.925	0.017	0.095	0.155

SMS: skin mottling score; MAP: mean arterial pressure; UO: urine volume; ΔLactate: lactate change over 2 h; CRT: capillary refill time; ScvO_2_: central venous oxygen saturation; VIS: vasoactive inotropic score; ΔPCO_2_: venous-to-arterial carbon dioxide partial pressure difference; OR: odd ratio; ρ: correlation coefficient

### Skin mottling and mortality risk

**Fig. 2:**
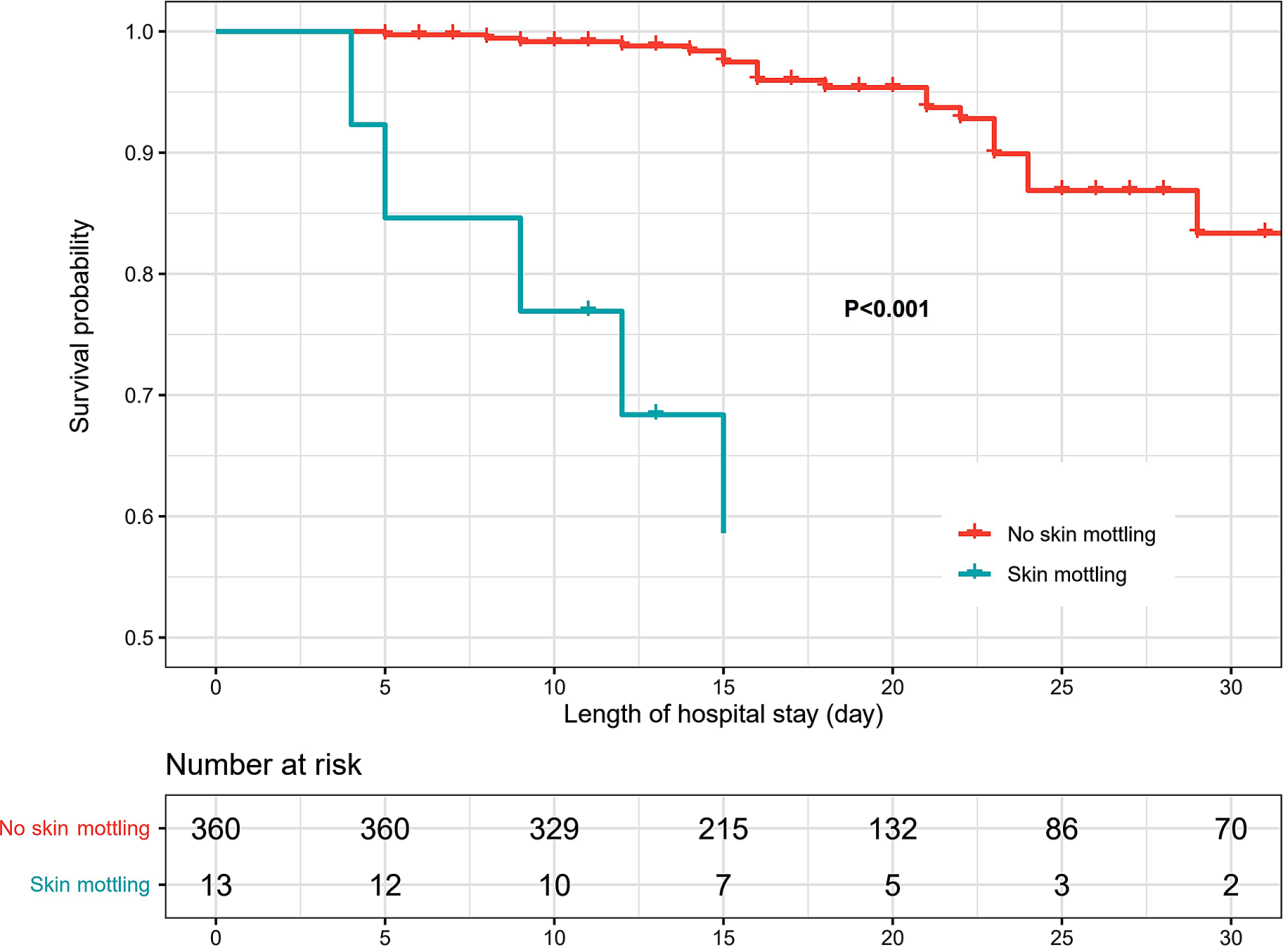
The survival curves for patients with and without skin mottling

The hospital mortality of overall patients was 14.7%, but it differed significantly between patients with and without skin mottling (84.6% vs. 12.2%, *p* < 0.001). In terms of survival curve, patients with skin mottling had a much worse survival (Fig. 2). In addition, the mortality risks were significantly higher in hypotension and shock populations, reaching 21.3% and 37.3%. For the overall population, the relative risk of skin mottling for mortality was 6.9 (95% CI: 4.8–9.9) and the odds ratio (OR) of SMS for mortality was 3.74 (95 CI: 1.61–8.65). The AUROCs of SMS for predicting hospital mortality were 0.60 (95 CI: 0.55–0.65), 0.63 (95 CI: 0.56–0.70) and 0.71 (95 CI: 0.57–0.83) in the overall, hypotension and shock populations, respectively (Table 1**and** Fig. 1.**C**). The optimal thresholds identified by the Youden index were all 1, corresponding to specificities of nearly 100, but a low sensitivity of 23 in the overall population, 26 in the hypotension population, and only 42 even in the shock population (Table 1).

## Discussion

The majority of our understanding of SMS comes from studies involving patients with sepsis. However, those who underwent cardiac surgery exhibit distinct hemodynamics from patients with sepsis. This study represented the first investigation of the application value of SMS in critically ill cardiac surgical patients and demonstrated that SMS was a highly specific yet less sensitive parameter of peripheral tissue hypoperfusion in these patients.

There is a lack of a gold standard for the assessment of tissue hypoperfusion, but at present a markedly prolonged CRT is generally regarded as a significant manifestation of peripheral tissue hypoperfusion [1]. Besides, to fully assess the value of SMS for patients undergoing cardiac surgery, we identified populations using three levels of criteria ranging from broad to strict. The first population is the largest in scope, and even patients with mild manifestations of possible hypoperfusion are included, in line with the need for universal screening for hypoperfusion. For such a population, the occurrence of skin mottling and the accuracy of SMS in identifying tissue hypoperfusion was not so good. In this population, although the occurrence of skin mottling and the overall accuracy and sensitivity of SMS in identifying tissue hypoperfusion were low, the specificity was very high. Fortunately, if the patients were restricted to more severe hypotension or shock, the accuracy and sensitivity of SMS would be significantly improved. This becomes a favorable reason to support the use of SMS to assess tissue perfusion in postoperative cardiac patients.

A recent study by Merdji,et al. has confirmed that the presence of skin mottling in patients with cardiogenic shock was an important prognosis marker [20]. We also found this phenomenon and SMS had a high specificity in predicting mortality risk. This study used data form a cohort of cardiac surgical patients who were deemed to be at high risk of tissue hypoperfusion. While these patients represented a minority, accounting for less than 10% of the overall cardiac surgical population, their hospital mortality was alarmingly high, at 15%. Of these patients, nearly half presented with hypotension, and one in seven met the criteria for shock, indicating varying degrees of tissue hypoperfusion risk. Our analysis revealed that the mortality for patients exhibiting any degree of skin mottling was considerably elevated. The optimal cut-off value for SMS was determined to be 1, which indicated that even minimal skin mottling conferred a significant risk of mortality. Furthermore, the specificity and PPV of SMS was found to be as very high. These findings suggest that any degree of skin mottling should be considered a critical warning sign in the postoperative period for cardiac surgical patients.

In patients with sepsis or septic shock, the presence of skin mottling was frequently observed, with reported occurrence ranging from 40 to 80% [9, 10, 21]. Coudroy et al. conducted an investigation in a medical ICU and found that the prevalence of skin mottling was 29% in overall patients and 49% in patients with septic shock [8]. In contrast, skin mottling appears to manifest later or to a lesser extent in non-septic populations. For instance, Ferly et al. reported a case of severe cardiogenic shock due to acute myocardial infarction with a SMS of only two, despite the presence of extreme circulatory collapse [22]. The occurrence of skin mottling was much lower in cardiac critically ill population observed in this study, when compared to patients with sepsis. Even in the population of shock, the occurrence was half or one-third of that observed in patients with septic shock. These findings suggest that there may be many severe patients who did not exhibit skin mottling, making the SMS less sensitive in detecting these patients.

Physiologically, the onset of circulatory failure differs between sepsis and cardiac critically ill patients, with the former affecting the microcirculation and the latter affecting the macro-circulation followed by involvement of the microcirculation [23]. As a result, septic patients have greater heterogeneity of capillary perfusion and function [23], which may account for the higher vulnerability to skin mottling in this population. It is worth noting, however, that the sensitivity of SMS was relatively low in our patient population. As such, it was advisable to utilize other tissue perfusion parameters in combination with SMS to compensate for this deficiency in clinical practice.

The current investigation has ascertained UO and CRT as the most significant factors that are linked to the presence or absence of skin mottling in critically ill patients who underwent cardiac surgery. UO is a reflection of visceral perfusion [11], while CRT is an indicator of peripheral tissue perfusion [1, 24]. The amalgamation of these two parameters is primarily responsible for the occurrence of skin mottling, with a substantial proportion of skin mottling patients displaying significant oliguria and prolonged CRT. This finding corroborates the fact that skin mottling is a sign of markedly inadequate tissue perfusion.

In clinical settings, our research provides crucial insights into the use of SMS among cardiac surgery patients. The detection of skin mottling serves as a significant predictor of increased mortality risk. As a marker of tissue perfusion, SMS is easily observed with a brief visual inspection, particularly effective in identifying severe hypoperfusion. The prompt recognition and reporting of skin mottling by bedside nurses are imperative for timely patient evaluation and necessary interventions. For clinicians, a quick examination for the presence and severity of skin mottling on the patient’s legs can be conducted during shift handovers, requiring only a few seconds. Recognizing skin mottling is vital as it may necessitate the initiation of hemodynamic optimization strategies to enhance tissue perfusion.

### Study limitations

Our study had several limitations. Firstly, the study was conducted at a single cardiac center, albeit one with a high patient volume. Future investigations may be necessary to validate our findings in other cardiac centers to ensure the generalizability of the results. Secondly, although SMS was evaluated once for each patient, we did not assess the prognostic impacts of changes in SMS following interventions. Therefore, the potential impact of dynamic changes in SMS on patient outcomes remains unclear. Thirdly, the proportion of patients occurring skin mottling was lower in our population compared to sepsis, and further research is warranted to elucidate the underlying mechanisms responsible for this difference. Fourth, patients with mechanical circulatory support, the most severe individuals in cardiac surgery population, were excluded because femoral artery cannulation directly affects blood flow of lower limbs. Finally, we did not collect hemodynamic parameters such as cardiac output, which may provide additional insights into the pathophysiology of skin mottling. Future investigations incorporating such data may aid in enhancing our understanding of skin mottling in the context of cardiogenic hemodynamic profiles.

## Conclusion

Among cardiac surgery patients, SMS demonstrated moderate accuracy in detecting peripheral tissue hypoperfusion, with high specificity but relatively lower sensitivity. Regardless of other clinical presentation, the presence of skin mottling of any size should be alarming and require careful assessment by the physician.

## Data Availability

The data sets used and analyzed during the current study are available from the corresponding author upon reasonable request.

## Abbreviations

SMS: Skin mottling score
CRT: Capillary refill time
SBP: Systolic blood pressure
DBP: Diastolic blood pressure
MAP: Mean arterial pressure
PP: Pulse pressure
HR: Heart rate
UO: Urine output
ΔPCO_2_: Venous-to-arterial carbon dioxide partial pressure difference
VIS: Vasoactive inotropic score
ScvO_2_: Central venous oxygen saturation
PPV: positive predictive value
NPV: negative predictive value
AUROC: Area under receiver operator characteristic
